# 3-(2-Bromo­phenyl­sulfon­yl)-5-cyclo­hexyl-2-methyl-1-benzo­furan

**DOI:** 10.1107/S1600536814003547

**Published:** 2014-02-22

**Authors:** Hong Dae Choi, Pil Ja Seo, Uk Lee

**Affiliations:** a Department of Chemistry, Dongeui University, San 24 Kaya-dong, Busanjin-gu, Busan 614-714, Republic of Korea; b Department of Chemistry, Pukyong National University, 599-1 Daeyeon 3-dong, Nam-gu, Busan 608-737, Republic of Korea

## Abstract

In the title compound, C_21_H_21_BrO_3_S, the cyclo­hexyl ring adopts a chair conformation. The dihedral angle between the mean planes of the benzo­furan and 2-bromo­phenyl fragments is 82.47 (5)°. In the crystal, mol­ecules related by inversion are paired into dimers *via* C—H⋯π and π–π inter­actions, the latter are indicated by the short distance of 3.607 (3) Å between the centroids of the furan rings. Inter­molecular C—H⋯O hydrogen bonds and short Br⋯O [3.280 (1) Å] contacts further consolidate the crystal packing.

## Related literature   

For background information and the crystal structures of related compounds, see: Choi *et al.* (2011 ▶, 2012*a* ▶,*b* ▶). For a review of halogen bonding, see: Politzer *et al.* (2007 ▶).
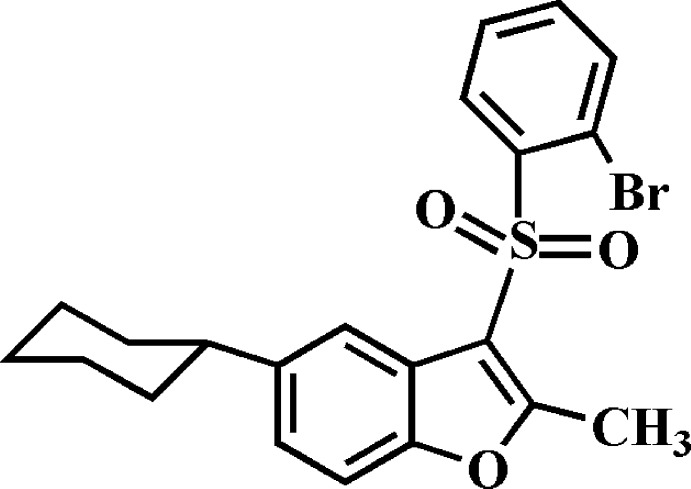


## Experimental   

### 

#### Crystal data  

C_21_H_21_BrO_3_S
*M* *_r_* = 433.35
Monoclinic, 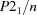

*a* = 7.3548 (1) Å
*b* = 20.4554 (4) Å
*c* = 12.6801 (2) Å
β = 92.463 (1)°
*V* = 1905.90 (5) Å^3^
*Z* = 4
Mo *K*α radiation
μ = 2.28 mm^−1^
*T* = 173 K
0.46 × 0.35 × 0.23 mm

#### Data collection  

Bruker SMART APEXII CCD diffractometer
Absorption correction: multi-scan (*SADABS*; Bruker, 2009 ▶) *T*_min_ = 0.507, *T*_max_ = 0.746
19277 measured reflections
4748 independent reflections
4028 reflections with *I* > 2σ(*I*)
*R*_int_ = 0.031

#### Refinement  

*R*[*F* ^2^ > 2σ(*F* ^2^)] = 0.031
*wR*(*F* ^2^) = 0.083
*S* = 1.03
4748 reflections
235 parameters
H-atom parameters constrained
Δρ_max_ = 0.60 e Å^−3^
Δρ_min_ = −0.56 e Å^−3^

### 

Data collection: *APEX2* (Bruker, 2009 ▶); cell refinement: *SAINT* (Bruker, 2009 ▶); data reduction: *SAINT*; program(s) used to solve structure: *SHELXS97* (Sheldrick, 2008 ▶); program(s) used to refine structure: *SHELXL97* (Sheldrick, 2008 ▶); molecular graphics: *ORTEP-3 for Windows* (Farrugia, 2012 ▶) and *DIAMOND* (Brandenburg, 1998 ▶); software used to prepare material for publication: *SHELXL97*.

## Supplementary Material

Crystal structure: contains datablock(s) I. DOI: 10.1107/S1600536814003547/cv5444sup1.cif

Structure factors: contains datablock(s) I. DOI: 10.1107/S1600536814003547/cv5444Isup2.hkl

Supporting information file. DOI: 10.1107/S1600536814003547/cv5444Isup3.cml

CCDC reference: 987378

Additional supporting information:  crystallographic information; 3D view; checkCIF report

## Figures and Tables

**Table 1:** Hydrogen-bond geometry (Å, °) *Cg*1 is the centroid of the C2–C7 benzene ring.

*D*—H⋯*A*	*D*—H	H⋯*A*	*D*⋯*A*	*D*—H⋯*A*
C19—H19⋯O2^i^	0.95	2.61	3.487 (2)	155
C20—H20⋯O3^i^	0.95	2.56	3.382 (2)	144
C15—H15*C*⋯*Cg*1^ii^	0.98	2.69	3.531 (2)	144

Symmetry codes: (i) 
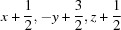
; (ii) 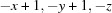.

**Fig. 1.**
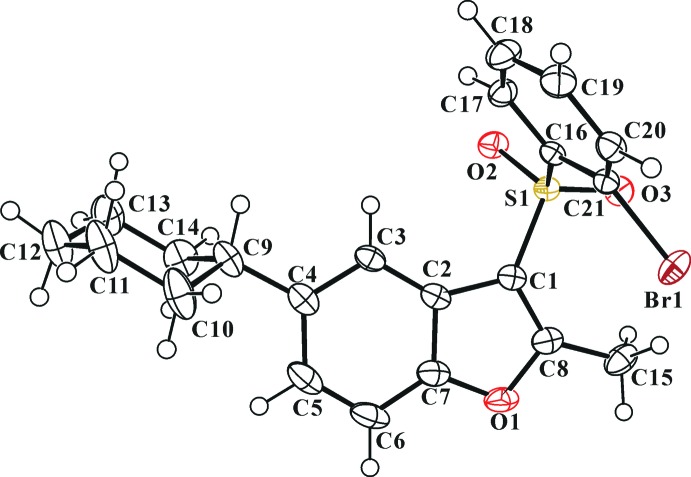
The molecular structure of the title compound with the atom numbering scheme. Displacement ellipsoids are drawn at the 50% probability level. H atoms are presented as small spheres of arbitrary radius.

**Fig. 2.**
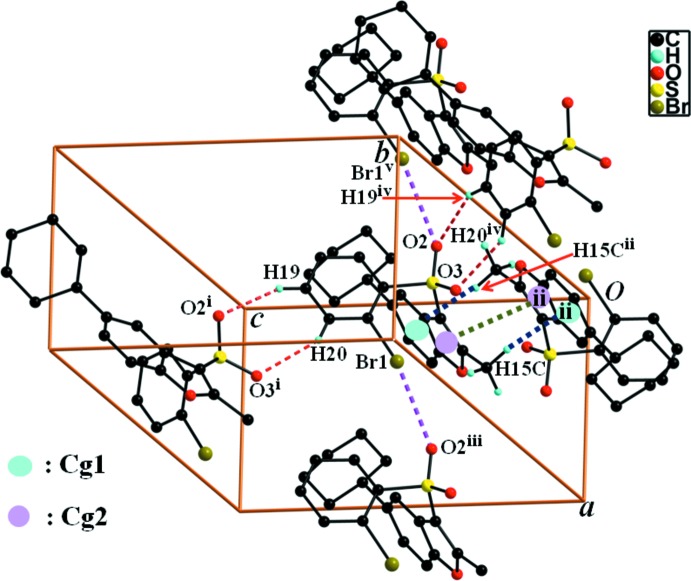
A view of the C—H···O, π···π, C—H···π and Br···O interactions (dashed lines) in the crystal structure of the title compound. H atoms non-participating in hydrogen-bonding were omitted for clarity. [Symmetry codes : (i) x + 1/2, - y + 3/2, z + 1/2; (ii) - x + 1, - y + 1, - z; (iii) x + 1, y, z; (iv) x - 1/2, - y + 3/2, z - 1/2 ; (v) x - 1, y, z.]

## supplementary crystallographic information

### 1. Comment

As a part of our continuing study of 5-cyclohexyl-2-methyl-1-benzofuran derivatives containing 4-fluorophenylsulfonyl (Choi *et al.*, 2011), 4-bromophenylsulfonyl (Choi *et al.*, 2012*a*) and 4-methylphenylsulfonyl (Choi *et al.*, 2012*b*) substituents in 3-position, we report herein the crystal structure of the title compound.

In the title molecule (Fig. 1), the cyclohexyl ring adopts a chair conformation. The benzofuran ring system is essentially planar, with a mean deviation of 0.009 (1) Å from the least-squares plane defined by the nine constituent atoms. The 2-bromophenyl ring is essentially planar, with a mean deviation of 0.004 (1) Å from the least-squares plane defined by the six constituent atoms. The dihedral angle formed by the benzofuran ring system and the 2-bromophenyl ring is 82.47 (5)°.

In the crystal structure (Fig. 2), the molecules related by inversion are paired into dimers *via* C—H···π (Table 1, Cg1 is the centroid of the C2-C7 benzene ring) and π···π interactions, the latter are proved by short distance of 3.607 (3) Å between the centroids of furan rings (Cg2 is the centroid of the C1/C2/C7/O1/C8 furan ring). These dimers are further packed by intermolecular C—H···O hydrogen bonds (Table 1) and short Br···O halogen-bondings (Politzer *et al.*, 2007) between the bromine atom and the oxygen atom of the O═S═O unit [Br1···O2^iii^ = 3.280 (1) Å, C21—Br1···O2^iii^ = 157.45 (5)°; symmerty code: (iii) x+1, y, z].

### 2. Experimental

3-Chloroperoxybenzoic acid (77%, 426 mg, 1.9 mmol) was added in small portions to a stirred solution of 3-(2-bromophenylsulfanyl)-5-cyclohexyl-2-methyl-1-benzofuran (361 mg, 0.9 mmol) in dichloromethane (40 mL) at 273 K. After being stirred at room temperature for 8h, the mixture was washed with saturated sodium bicarbonate solution and the organic layer was separated, dried over magnesium sulfate, filtered and concentrated at reduced pressure. The residue was purified by column chromatography (benzene) to afford the title compound as a colourless solid [yield 76%, m.p. 445–446 K; *R*_f_ = 0.51 (benzene)]. Single crystals suitable for X-ray diffraction were prepared by slow evaporation of a solution of the title compound in ethyl acetate at room temperature.

### 3. Refinement

All H atoms were positioned geometrically and refined using a riding model, with C—H = 0.95 Å for aryl, 1.00 Å for methine, 0.99 Å for methylene and 0.98 Å for methyl H atoms, respectively. *U*_iso_ (H) = 1.2*U*_eq_ (C) for aryl, methine and methylene, and 1.5*U*_eq_ (C) for methyl H atoms.

### Crystal data

**Table:**

C_21_H_21_BrO_3_S	*F*(000) = 888
*M**_r_* = 433.35	*D*_x_ = 1.510 Mg m^−^^3^
Monoclinic, *P*2_1_/*n*	Melting point = 445–446 K
Hall symbol: -P 2yn	Mo *K*α radiation, λ = 0.71073 Å
*a* = 7.3548 (1) Å	Cell parameters from 7682 reflections
*b* = 20.4554 (4) Å	θ = 2.6–28.2°
*c* = 12.6801 (2) Å	µ = 2.28 mm^−^^1^
β = 92.463 (1)°	*T* = 173 K
*V* = 1905.90 (5) Å^3^	Block, colourless
*Z* = 4	0.46 × 0.35 × 0.23 mm

### Data collection

**Table:**

Bruker SMART APEXII CCD diffractometer	4748 independent reflections
Radiation source: rotating anode	4028 reflections with *I* > 2σ(*I*)
Graphite multilayer monochromator	*R*_int_ = 0.031
Detector resolution: 10.0 pixels mm^-1^	θ_max_ = 28.4°, θ_min_ = 1.9°
φ and ω scans	*h* = −8→9
Absorption correction: multi-scan (*SADABS*; Bruker, 2009)	*k* = −27→21
*T*_min_ = 0.507, *T*_max_ = 0.746	*l* = −16→16
19277 measured reflections	

### Refinement

**Table:**

Refinement on *F*^2^	Primary atom site location: structure-invariant direct methods
Least-squares matrix: full	Secondary atom site location: difference Fourier map
*R*[*F*^2^ > 2σ(*F*^2^)] = 0.031	Hydrogen site location: difference Fourier map
*wR*(*F*^2^) = 0.083	H-atom parameters constrained
*S* = 1.03	*w* = 1/[σ^2^(*F*_o_^2^) + (0.0428*P*)^2^ + 1.0558*P*] where *P* = (*F*_o_^2^ + 2*F*_c_^2^)/3
4748 reflections	(Δ/σ)_max_ = 0.001
235 parameters	Δρ_max_ = 0.60 e Å^−^^3^
0 restraints	Δρ_min_ = −0.56 e Å^−^^3^

### Special details

**Table:**

Geometry. All esds (except the esd in the dihedral angle between two l.s. planes) are estimated using the full covariance matrix. The cell esds are taken into account individually in the estimation of esds in distances, angles and torsion angles; correlations between esds in cell parameters are only used when they are defined by crystal symmetry. An approximate (isotropic) treatment of cell esds is used for estimating esds involving l.s. planes.
Refinement. Refinement of F^2^ against ALL reflections. The weighted R-factor wR and goodness of fit S are based on F^2^, conventional R-factors R are based on F, with F set to zero for negative F^2^. The threshold expression of F^2^ > 2sigma(F^2^) is used only for calculating R-factors(gt) etc. and is not relevant to the choice of reflections for refinement. R-factors based on F^2^ are statistically about twice as large as those based on F, and R- factors based on ALL data will be even larger.

### Fractional atomic coordinates and isotropic or equivalent isotropic displacement parameters (Å^2^)

**Table:**

	*x*	*y*	*z*	*U*_iso_*/*U*_eq_	
Br1	0.86740 (3)	0.705237 (11)	0.155454 (16)	0.02977 (8)	
S1	0.43880 (6)	0.64983 (2)	0.08850 (3)	0.01954 (10)	
O1	0.75164 (19)	0.49455 (7)	0.08436 (13)	0.0321 (3)	
O2	0.24696 (18)	0.63578 (7)	0.08776 (11)	0.0263 (3)	
O3	0.5080 (2)	0.68541 (7)	0.00109 (11)	0.0277 (3)	
C1	0.5550 (3)	0.57650 (9)	0.10478 (15)	0.0216 (4)	
C2	0.5104 (3)	0.52574 (9)	0.17993 (15)	0.0226 (4)	
C3	0.3820 (3)	0.51623 (9)	0.25675 (15)	0.0238 (4)	
H3	0.2955	0.5493	0.2706	0.029*	
C4	0.3828 (3)	0.45752 (10)	0.31282 (16)	0.0277 (4)	
C5	0.5130 (3)	0.40942 (11)	0.2906 (2)	0.0370 (5)	
H5	0.5123	0.3694	0.3287	0.044*	
C6	0.6409 (3)	0.41813 (10)	0.2159 (2)	0.0374 (5)	
H6	0.7278	0.3853	0.2018	0.045*	
C7	0.6369 (3)	0.47670 (10)	0.16263 (17)	0.0283 (4)	
C8	0.6990 (3)	0.55528 (10)	0.05010 (17)	0.0266 (4)	
C9	0.2428 (3)	0.44470 (10)	0.39452 (17)	0.0315 (5)	
H9	0.1781	0.4869	0.4066	0.038*	
C10	0.3273 (4)	0.42243 (13)	0.50155 (19)	0.0449 (7)	
H10A	0.3927	0.3806	0.4924	0.054*	
H10B	0.4167	0.4554	0.5279	0.054*	
C11	0.1817 (4)	0.41342 (13)	0.5820 (2)	0.0480 (7)	
H11A	0.2389	0.3973	0.6492	0.058*	
H11B	0.1242	0.4561	0.5960	0.058*	
C12	0.0365 (4)	0.36519 (13)	0.5428 (2)	0.0476 (7)	
H12A	−0.0608	0.3631	0.5943	0.057*	
H12B	0.0911	0.3211	0.5381	0.057*	
C13	−0.0463 (3)	0.38442 (13)	0.4352 (2)	0.0418 (6)	
H13A	−0.1179	0.4251	0.4422	0.050*	
H13B	−0.1302	0.3495	0.4093	0.050*	
C14	0.1001 (3)	0.39514 (12)	0.35539 (18)	0.0376 (5)	
H14A	0.0423	0.4108	0.2881	0.045*	
H14B	0.1606	0.3530	0.3414	0.045*	
C15	0.8082 (3)	0.58153 (12)	−0.03582 (18)	0.0338 (5)	
H15A	0.9024	0.5498	−0.0529	0.051*	
H15B	0.8657	0.6226	−0.0129	0.051*	
H15C	0.7287	0.5895	−0.0985	0.051*	
C16	0.4887 (2)	0.69432 (8)	0.20708 (14)	0.0183 (4)	
C17	0.3458 (3)	0.70484 (9)	0.27279 (16)	0.0247 (4)	
H17	0.2291	0.6872	0.2547	0.030*	
C18	0.3719 (3)	0.74109 (11)	0.36512 (17)	0.0309 (5)	
H18	0.2732	0.7486	0.4095	0.037*	
C19	0.5423 (3)	0.76600 (11)	0.39179 (17)	0.0294 (4)	
H19	0.5603	0.7908	0.4547	0.035*	
C20	0.6873 (3)	0.75516 (9)	0.32758 (16)	0.0238 (4)	
H20	0.8044	0.7720	0.3468	0.029*	
C21	0.6602 (2)	0.71964 (9)	0.23533 (15)	0.0199 (4)	

### Atomic displacement parameters (Å^2^)

**Table:**

	*U*^11^	*U*^22^	*U*^33^	*U*^12^	*U*^13^	*U*^23^
Br1	0.01868 (11)	0.04310 (14)	0.02774 (12)	−0.00582 (8)	0.00347 (7)	−0.00653 (9)
S1	0.0179 (2)	0.0223 (2)	0.0181 (2)	0.00098 (17)	−0.00338 (16)	−0.00055 (17)
O1	0.0245 (7)	0.0260 (7)	0.0458 (9)	0.0041 (6)	0.0011 (6)	−0.0110 (7)
O2	0.0184 (6)	0.0298 (7)	0.0302 (7)	0.0002 (5)	−0.0059 (5)	−0.0027 (6)
O3	0.0303 (7)	0.0325 (7)	0.0201 (7)	0.0014 (6)	−0.0016 (5)	0.0038 (6)
C1	0.0216 (9)	0.0209 (9)	0.0220 (9)	0.0001 (7)	−0.0043 (7)	−0.0052 (7)
C2	0.0234 (9)	0.0187 (9)	0.0248 (9)	0.0003 (7)	−0.0081 (7)	−0.0037 (7)
C3	0.0265 (10)	0.0192 (9)	0.0252 (9)	0.0020 (7)	−0.0057 (7)	−0.0022 (8)
C4	0.0329 (11)	0.0216 (9)	0.0275 (10)	−0.0027 (8)	−0.0095 (8)	0.0005 (8)
C5	0.0410 (13)	0.0195 (10)	0.0493 (14)	0.0003 (9)	−0.0127 (11)	0.0044 (10)
C6	0.0340 (12)	0.0202 (10)	0.0568 (15)	0.0068 (9)	−0.0104 (10)	−0.0058 (10)
C7	0.0235 (10)	0.0226 (10)	0.0382 (12)	0.0012 (8)	−0.0054 (8)	−0.0080 (9)
C8	0.0219 (9)	0.0268 (10)	0.0306 (10)	−0.0007 (8)	−0.0030 (8)	−0.0100 (8)
C9	0.0426 (12)	0.0226 (10)	0.0285 (10)	−0.0037 (9)	−0.0075 (9)	0.0061 (8)
C10	0.0571 (16)	0.0402 (13)	0.0355 (13)	−0.0230 (12)	−0.0200 (11)	0.0145 (11)
C11	0.0709 (18)	0.0395 (14)	0.0323 (12)	−0.0198 (13)	−0.0125 (12)	0.0155 (11)
C12	0.0569 (16)	0.0409 (14)	0.0441 (14)	−0.0172 (12)	−0.0077 (12)	0.0185 (12)
C13	0.0422 (13)	0.0407 (13)	0.0417 (13)	−0.0104 (11)	−0.0076 (10)	0.0078 (11)
C14	0.0373 (12)	0.0395 (13)	0.0350 (12)	−0.0069 (10)	−0.0093 (10)	−0.0001 (10)
C15	0.0268 (10)	0.0424 (13)	0.0327 (11)	−0.0029 (9)	0.0062 (8)	−0.0138 (10)
C16	0.0193 (9)	0.0164 (8)	0.0189 (8)	0.0017 (7)	−0.0020 (7)	0.0006 (7)
C17	0.0196 (9)	0.0269 (10)	0.0277 (10)	−0.0004 (7)	0.0019 (7)	−0.0016 (8)
C18	0.0290 (11)	0.0335 (11)	0.0307 (11)	0.0017 (9)	0.0071 (8)	−0.0079 (9)
C19	0.0351 (11)	0.0274 (10)	0.0258 (10)	−0.0004 (9)	0.0019 (8)	−0.0082 (8)
C20	0.0236 (9)	0.0207 (9)	0.0267 (9)	−0.0021 (7)	−0.0041 (7)	−0.0013 (8)
C21	0.0192 (9)	0.0188 (9)	0.0217 (9)	0.0012 (7)	0.0005 (7)	0.0019 (7)

### Geometric parameters (Å, º)

**Table:**

Br1—C21	1.8887 (19)	C10—H10B	0.9900
Br1—O2^i^	3.2793 (14)	C11—C12	1.521 (3)
S1—O3	1.4372 (15)	C11—H11A	0.9900
S1—O2	1.4395 (14)	C11—H11B	0.9900
S1—C1	1.7342 (19)	C12—C13	1.522 (3)
S1—C16	1.7823 (19)	C12—H12A	0.9900
O1—C8	1.367 (3)	C12—H12B	0.9900
O1—C7	1.379 (3)	C13—C14	1.525 (3)
C1—C8	1.361 (3)	C13—H13A	0.9900
C1—C2	1.456 (3)	C13—H13B	0.9900
C2—C7	1.392 (3)	C14—H14A	0.9900
C2—C3	1.399 (3)	C14—H14B	0.9900
C3—C4	1.395 (3)	C15—H15A	0.9800
C3—H3	0.9500	C15—H15B	0.9800
C4—C5	1.410 (3)	C15—H15C	0.9800
C4—C9	1.515 (3)	C16—C17	1.386 (3)
C5—C6	1.374 (4)	C16—C21	1.396 (3)
C5—H5	0.9500	C17—C18	1.392 (3)
C6—C7	1.375 (3)	C17—H17	0.9500
C6—H6	0.9500	C18—C19	1.381 (3)
C8—C15	1.482 (3)	C18—H18	0.9500
C9—C14	1.527 (3)	C19—C20	1.387 (3)
C9—C10	1.537 (3)	C19—H19	0.9500
C9—H9	1.0000	C20—C21	1.384 (3)
C10—C11	1.522 (4)	C20—H20	0.9500
C10—H10A	0.9900		
			
C21—Br1—O2^i^	157.43 (6)	C10—C11—H11A	109.4
O3—S1—O2	118.40 (8)	C12—C11—H11B	109.4
O3—S1—C1	109.92 (9)	C10—C11—H11B	109.4
O2—S1—C1	107.81 (9)	H11A—C11—H11B	108.0
O3—S1—C16	108.97 (9)	C11—C12—C13	111.9 (2)
O2—S1—C16	105.89 (9)	C11—C12—H12A	109.2
C1—S1—C16	104.99 (8)	C13—C12—H12A	109.2
C8—O1—C7	107.19 (16)	C11—C12—H12B	109.2
C8—C1—C2	107.91 (17)	C13—C12—H12B	109.2
C8—C1—S1	127.25 (16)	H12A—C12—H12B	107.9
C2—C1—S1	124.83 (15)	C12—C13—C14	111.4 (2)
C7—C2—C3	119.02 (18)	C12—C13—H13A	109.3
C7—C2—C1	103.90 (18)	C14—C13—H13A	109.3
C3—C2—C1	137.08 (18)	C12—C13—H13B	109.3
C4—C3—C2	119.10 (18)	C14—C13—H13B	109.3
C4—C3—H3	120.5	H13A—C13—H13B	108.0
C2—C3—H3	120.5	C13—C14—C9	112.0 (2)
C3—C4—C5	119.1 (2)	C13—C14—H14A	109.2
C3—C4—C9	120.63 (19)	C9—C14—H14A	109.2
C5—C4—C9	120.23 (19)	C13—C14—H14B	109.2
C6—C5—C4	122.5 (2)	C9—C14—H14B	109.2
C6—C5—H5	118.7	H14A—C14—H14B	107.9
C4—C5—H5	118.7	C8—C15—H15A	109.5
C5—C6—C7	116.8 (2)	C8—C15—H15B	109.5
C5—C6—H6	121.6	H15A—C15—H15B	109.5
C7—C6—H6	121.6	C8—C15—H15C	109.5
C6—C7—O1	125.7 (2)	H15A—C15—H15C	109.5
C6—C7—C2	123.4 (2)	H15B—C15—H15C	109.5
O1—C7—C2	110.87 (18)	C17—C16—C21	119.25 (17)
C1—C8—O1	110.13 (18)	C17—C16—S1	116.89 (14)
C1—C8—C15	135.8 (2)	C21—C16—S1	123.85 (15)
O1—C8—C15	114.07 (18)	C16—C17—C18	120.49 (18)
C4—C9—C14	111.76 (18)	C16—C17—H17	119.8
C4—C9—C10	113.20 (19)	C18—C17—H17	119.8
C14—C9—C10	109.66 (18)	C19—C18—C17	119.56 (19)
C4—C9—H9	107.3	C19—C18—H18	120.2
C14—C9—H9	107.3	C17—C18—H18	120.2
C10—C9—H9	107.3	C18—C19—C20	120.64 (19)
C11—C10—C9	111.0 (2)	C18—C19—H19	119.7
C11—C10—H10A	109.4	C20—C19—H19	119.7
C9—C10—H10A	109.4	C21—C20—C19	119.59 (18)
C11—C10—H10B	109.4	C21—C20—H20	120.2
C9—C10—H10B	109.4	C19—C20—H20	120.2
H10A—C10—H10B	108.0	C20—C21—C16	120.47 (18)
C12—C11—C10	111.3 (2)	C20—C21—Br1	116.43 (14)
C12—C11—H11A	109.4	C16—C21—Br1	123.06 (14)
			
O3—S1—C1—C8	4.1 (2)	C3—C4—C9—C14	106.7 (2)
O2—S1—C1—C8	134.53 (17)	C5—C4—C9—C14	−71.7 (2)
C16—S1—C1—C8	−112.91 (18)	C3—C4—C9—C10	−128.9 (2)
O3—S1—C1—C2	−174.52 (15)	C5—C4—C9—C10	52.7 (3)
O2—S1—C1—C2	−44.14 (17)	C4—C9—C10—C11	177.3 (2)
C16—S1—C1—C2	68.42 (17)	C14—C9—C10—C11	−57.2 (3)
C8—C1—C2—C7	−0.1 (2)	C9—C10—C11—C12	56.7 (3)
S1—C1—C2—C7	178.84 (14)	C10—C11—C12—C13	−54.5 (3)
C8—C1—C2—C3	−179.6 (2)	C11—C12—C13—C14	53.1 (3)
S1—C1—C2—C3	−0.7 (3)	C12—C13—C14—C9	−54.6 (3)
C7—C2—C3—C4	−0.9 (3)	C4—C9—C14—C13	−177.32 (19)
C1—C2—C3—C4	178.6 (2)	C10—C9—C14—C13	56.3 (3)
C2—C3—C4—C5	0.0 (3)	O3—S1—C16—C17	129.89 (15)
C2—C3—C4—C9	−178.36 (17)	O2—S1—C16—C17	1.52 (17)
C3—C4—C5—C6	0.4 (3)	C1—S1—C16—C17	−112.40 (15)
C9—C4—C5—C6	178.8 (2)	O3—S1—C16—C21	−48.82 (18)
C4—C5—C6—C7	0.0 (3)	O2—S1—C16—C21	−177.19 (15)
C5—C6—C7—O1	−179.2 (2)	C1—S1—C16—C21	68.89 (17)
C5—C6—C7—C2	−0.9 (3)	C21—C16—C17—C18	1.0 (3)
C8—O1—C7—C6	178.2 (2)	S1—C16—C17—C18	−177.78 (16)
C8—O1—C7—C2	−0.4 (2)	C16—C17—C18—C19	−0.8 (3)
C3—C2—C7—C6	1.3 (3)	C17—C18—C19—C20	−0.1 (3)
C1—C2—C7—C6	−178.32 (19)	C18—C19—C20—C21	0.7 (3)
C3—C2—C7—O1	179.89 (16)	C19—C20—C21—C16	−0.5 (3)
C1—C2—C7—O1	0.3 (2)	C19—C20—C21—Br1	−178.13 (15)
C2—C1—C8—O1	−0.2 (2)	C17—C16—C21—C20	−0.4 (3)
S1—C1—C8—O1	−179.02 (13)	S1—C16—C21—C20	178.32 (14)
C2—C1—C8—C15	179.3 (2)	C17—C16—C21—Br1	177.12 (14)
S1—C1—C8—C15	0.5 (3)	S1—C16—C21—Br1	−4.2 (2)
C7—O1—C8—C1	0.3 (2)	O2^i^—Br1—C21—C20	80.7 (2)
C7—O1—C8—C15	−179.30 (17)	O2^i^—Br1—C21—C16	−96.9 (2)

Symmetry code: (i) *x*+1, *y*, *z*.

### Hydrogen-bond geometry (Å, º)

Cg1 is the centroid of the C2–C7 benzene ring.

**Table:**

*D*—H···*A*	*D*—H	H···*A*	*D*···*A*	*D*—H···*A*
C19—H19···O2^ii^	0.95	2.61	3.487 (2)	155
C20—H20···O3^ii^	0.95	2.56	3.382 (2)	144
C15—H15*C*···*Cg*1^iii^	0.98	2.69	3.531 (2)	144

Symmetry codes: (ii) *x*+1/2, −*y*+3/2, *z*+1/2; (iii) −*x*+1, −*y*+1, −*z*.
